# Identification of chironomid species as natural reservoirs of toxigenic *Vibrio cholerae* strains with pandemic potential

**DOI:** 10.1371/journal.pntd.0008959

**Published:** 2020-12-23

**Authors:** Sivan Laviad-Shitrit, Rotem Sela, Leena Thorat, Yehonatan Sharaby, Ido Izhaki, Bimalendu B. Nath, Malka Halpern

**Affiliations:** 1 Department of Evolutionary and Environmental Biology, University of Haifa, Haifa, Israel; 2 Department of Zoology, Savitribai Phule Pune University, Pune, India; 3 Department of Biology, York University, Toronto, Canada; 4 Department of Biology and Environment, University of Haifa, Oranim, Tivon, Israel; Lowell General Hospital, UNITED STATES

## Abstract

*Vibrio cholerae* causes the fatal cholera diarrhea. Chironomids (*Diptera; Chironomidae*) are abundant in freshwater aquatic habitats and estuaries and are natural reservoirs of *V*. *cholerae*. Until now, only the non-O1/O139 serogroups of *V*. *cholerae* were identified in chironomids. Here, we explored whether chironomids are natural reservoirs of *V*. *cholerae* O1/O139 serogroups, which are associated with cholera endemics and pandemics. All four life stages of chironomids were sampled from two rivers, and a laboratory culture in Pune, India, and from a pond in Israel. In total, we analyzed 223 chironomid samples. The presence of *V*. *cholerae* O1/O139 serogroups was verified using molecular tools. Nine chironomid species were identified; of them, *Chironomus circumdatus* was the most abundant. The presence of *V*. *cholerae* serogroup O1 and the cholera toxin genes were detected in samples from all chironomid species. However, serogroup O139 was detected in only two chironomid species. Besides PCR to detect specific genes, a metagenomic analysis that was performed in three selected *C*. *ramosus* larvae, identified a list of virulence genes associated with *V*. *cholerae*. The findings provide evidence that chironomids are natural reservoirs of toxigenic *V*. *cholerae* O1/O139. Chironomid populations and *V*. *cholerae* show biannual peak patterns. A similar pattern is found for cholera epidemics in the Bengal Delta region. Thus, we hypothesize that monitoring chironomids in endemic areas of the disease may provide a novel tool for predicting and preventing cholera epidemics. Moreover, serogroup O139 was detected only in two chironomid species that have a restricted distribution in the Indian subcontinent, possibly explaining why the distribution of the O139 serogroup is limited.

## Author summary

*Vibrio cholerae*, is the causative agent of the diarrheal disease cholera, which infects millions of people worldwide. Despite intensive research, the environmental reservoir of *V*. *cholerae* is still unclear and under debate within the scientific community. Chironomids, an aquatic insect group that is closely related to mosquitoes, were reported to function as reservoirs of *V*. *cholerae*. Nevertheless, until now, there have been no reports regarding the presence of pandemic-causing *V*. *cholerae* O1/O139 serogroups in chironomids. By using molecular tools including metagenomic analyses of chironomids larval microbiomes, we provide here evidence for the first time, that chironomids harbor pandemic serogroups (O1/O139) of *V*. *cholerae*, which possess the cholera-toxin gene. Chironomid populations and *V*. *cholerae* demonstrate biannual peaks similar to the biannual peaks of cholera epidemics in the Bengal Delta region in India. Thus, we hypothesize that monitoring and controlling chironomid populations in endemic areas could be used as a tool for predicting and controlling the devastating cholera outbreaks.

## Introduction

*Vibrio cholerae*, is the causative agent of the diarrheal cholera disease which infects millions of people and causes 21,000 to 143,000 deaths, annually [1]. There are more than 200 *V*. *cholerae* serogroups; however, only serogroups O1 and O139 have been associated with cholera epidemics and pandemics [2]. Human infections occur mainly via the handling or consumption of contaminated food and water. Toxigenic strains pass through the human gastric acid barrier and colonize the epithelial cells of the small intestine. After colonization, the bacterium produces the cholera toxin which causes acute dehydration that if untreated–in many cases leads to death [2].

Chironomids (*Diptera; Chironomidae*), also known as non-biting midges, are globally distributed and one of the most abundant insects in freshwater habitats. Chironomids inhabit different water bodies: streams, rivers, lagoons, lakes, waste stabilization ponds, drinking water reservoirs, brackish water, estuaries, and marine environments [3]. Chironomids undergo a complete metamorphosis comprising the aquatic egg, larval, and pupal stages, while the terrestrial adults emerge into the air. Females lay egg masses on the water's edge. Each egg mass contains hundreds of eggs surrounded by a gelatinous matrix which is composed of glycoprotein and chitin [4–6].

Chironomids were found to be natural reservoirs of *V*. *cholerae* [7]. During the last 20 years, *V*. *cholerae* non-O1/O139 have been isolated from all chironomid life stages: egg masses [8–14], larvae [11,12,14,15], exuviae (empty pupal exoskeleton) [11,12], and adults [16].

Overall, 35 different *V*. *cholerae* non-O1/O139 serogroups were successfully identified from chironomids [17]. Most studies of the relationship between chironomids and *V*. *cholerae* were conducted in Israel, where the disease is not endemic. Halpern et al. [8] studied the presence of *V*. *cholerae* in chironomid egg masses that were sampled in India (Uttar Pradesh, West Bengal, and Maharashtra), Zanzibar, and Malawi; where 200 non-O1/O139 isolates were identified. Raz et al. [12] identified 326 *V*. *cholerae* non-O1/O139 isolates from egg masses, larvae, exuviae, and adults sampled in India. Lotfi et al. [11] isolated *V*. *cholerae* non-O1/O139 from *Chironomus transvaalensis* larvae and exuviae that were collected in Egypt.

It is assumed that different strains of *V*. *cholerae* non-O1/O139, as well as O1/O139 serogroups, inhabit the same habitats [17]. *V*. *cholerae* non-O1/O139 were isolated from the intestines of wild Little egrets (*Egretta garzetta*) and Black-crowned night herons (*Nycticorax nycticorax*). Using molecular tools, the presence of toxigenic *V*. *cholerae* O1 were confirmed in the intestine samples of the same individual birds from where *V*. *cholerae* non-O1/O139 were isolated [18]. Nevertheless, detecting toxigenic *V*. *cholerae* serogroups by isolation has been challenging because these strains are considerably less abundant compared to the non- pandemic strains that inhabit the same niche. In addition, the transition of *V*. *cholerae* into a viable but non-culturable (VBNC) state is another reason that toxigenic strains cannot be detected by traditional culture based methods [9,19,20]. In a survey of *V*. *cholerae* presence in fresh oysters in the USA, 14% of the samples contained *V*. *cholerae* non-O1 serogroups, whereas 0.9% of the samples contained the O1 serogroup [21].

Because hundreds of samples have to be screened to isolate toxigenic *V*. *cholerae* strains, numerous molecular techniques have been developed to screen for the presence of the toxigenic strains directly from the environmental samples e.g., the direct polymerase chain reaction (PCR) method to detect *V*. *cholerae* O1/O139 and the *ctxA* gene [22]; and the Cholera Sensitive Membrane Antigen Rapid Test (SMART), which includes immunoassay strips for the fast and direct detection of *V*. *cholerae* O1/O139 [23].

Our aim was to study whether chironomids are natural reservoirs of *V*. *cholerae* O1/O139, as was found for the non-O1/O139 serogroups [7]. For this, we sampled different life stages of chironomids from two rivers, and a laboratory culture in Pune, India, and from a waste stabilization pond in Israel; used molecular tools such as PCR for detection of specific genes; and performed a metagenomic analysis of the *V*. *cholerae* community associated with three selected *Chironomus* larvae. Understanding the environmental source of cholera epidemics is of high importance to allow predicting and controlling the spread of cholera outbreaks. In this context, the evidence presented in this paper confirms that chironomids serve as natural reservoirs not only for *V*. *cholerae* non-O1/O139 serogroups but also for the toxigenic O1/O139 serogroups which possess pandemic potential.

## Methods

### Chironomid sampling

Chironomids were sampled from four different sampling locations. Environmental samples from India were collected from two locations as described previously [24]: (i) Mula River, in the neighborhood of Holkar Bridge in the city of Pune, India (18.5551°N, 73.8618°E) and (ii) Mutha River, in the neighborhood of Vitthal Mandir in the city of Pune, India (18.2901°N, 73.4956°E) (iii) chironomid laboratory culture, which was originally collected from the Mula River sampling point in April 2018, and maintained under laboratory conditions for eight months (hereinafter laboratory culture, described below) (iv) Yokneam waste stabilization pond in northern Israel (hereinafter Yokneam) (32.6582°N, 35.1245°E). The samplings in India both in the rivers and the laboratory took place in November 2018. Chironomids were sampled in Israel in July 2018.

Environmental samples of egg masses, larvae, and, pupae were collected from plants (these developmental stages were found adhering to roots of water hyacinth, *Eichhornia crassipes*, [25]) (S1A and S1B Fig), and from the sediments at the river banks. Specifically, egg masses were collected gently with a pair of blunt forceps, and larvae and pupae were collected using scoops to gently lift the individuals from the sediments/substratum at the banks of the water bodies (larvae, S1C and S1D Fig). Adults were collected from hatching pupae. Hatching occurred 1–4 hours after samples were brought to the laboratory.

Overall, 223 samples were collected and analyzed from the four sampling sites [Mula River (n = 67); Mutha River (n = 51); Laboratory culture (n = 61); Yokneam, Israel (n = 44)]. All samples were brought to the laboratory immediately after collection. Each specimen was treated separately by washing and vortexing for one min in sterile water, and this procedure was repeated five times. This washing procedure was performed to remove any contamination or bacteria attached to the body of the insect. All samples were kept in 1.5 ml centrifuge tubes containing 750 μl of absolute ethanol and preserved at -20°C until DNA was extracted.

### Laboratory-culture maintenance

Larvae collected from the Mula river were validated for taxonomic identification using morphological and cytotaxonomic keys [26,27] and reared in non-toxic plastic tubs (Ø = 35 cm) containing sterilized beach sand at the bottom (S2A Fig). The rearing procedure was described previously [28,29]. In brief, to initiate rearing, ~100 late fourth instar larvae were transferred to a separate plastic tub. Culture tubs were flushed with fresh water every alternate day and were supplemented with 0.5 g of food (a mix of 5:1 by weight of *Sphagnum-*moss:yeast). When pupae were developed, the tubs were placed in a net cage to allow the adults' mating (S2B Fig). Emerging adults mated and laid fertile egg masses on the surface of the water. Egg masses were collected, transferred to hatching containers, and were maintained at 23±2°C, with a photoperiod of 14:10 hours of light:dark.

### DNA extraction

Before DNA was extracted, each specimen, which was kept in an ethanol tube, was centrifuged for 30 min at 10,000 rpm. Then, the ethanol was decanted. To remove the ethanol residue, the tubes were incubated for 10 min at 100°C on a heat block. Each sample was then washed with sterile saline water (0.85% NaCl) and crushed using a sterile homogenizer. DNA was extracted from all chironomid life stages using a DNA isolation kit (DNeasy Blood and Tissue, Qiagen, Germany), according to the manufacturer's instructions with minor modifications. Briefly, 180 μl of sterile enzymatic lysis buffer (20 mM Tris HCL pH 8, 2 mM sodium EDTA and 1.2% Triton-X-100) with 20 mg/ml lysozyme (Sigma-Aldrich, USA) was added to each sample, and the samples were incubated with shaking for 60 min at 37°C. Next, 25 μl proteinase K and 200 μl of Al buffer (Blood and Tissue, Qiagen, Germany), were added to each sample and the samples were incubated with shaking for 30 min at 56°C. Extraction then continued according to the manufacturer's instructions and DNA samples were stored at −20°C.

### Taxonomic identification of chironomid species

Taxonomic identification of chironomid species was performed by sequencing the *cytochrome oxidase* subunit I gene using PCR, as described before by Folmer et al. [30]. All sequences were compared to the available sequences in the NCBI GenBank database (https://www.ncbi.nlm.nih.gov/). Sequences were submitted to the GenBank database under accession numbers: MN934105-MN934321.

### Molecular detection of *Vibrio cholerae*

The presence of *V*. *cholerae* in the chironomid samples was detected using the *ompW* gene (an outer membrane protein, specific to *V*. *cholerae*). The presence of the cholera toxin was detected using the *ctxA* gene (cholera enterotoxin subunit A) [31]. In addition, all samples were tested for the presence of the toxigenic O1 and O139 serogroups, as described before by Rivera et al. [20]. The specific primers used for the identification of toxigenic serogroups, amplify specific genes involved in the biosynthesis of the surface O polysaccharides. The primers were VCO1F2 and VCO1R2, for the detection of O1 (*wbeO* gene; amplicon size 647 bp) [32], and VCO139F2 and VCO139R2, for the detection of O139 (amplicon size 741 bp) [20]. The PCR limit of detection (LOD) for the different genes was determined using *V*. *cholerae* O1 strain C6706. DNA was extracted from 1 ml of overnight bacterial cultures (in triplicates), as was described above. The colony-forming units per ml (cfu/ml) of the cultures were calculated for the triplicates and the LOD for *ompW*, *wbeO* and *ctxA* genes was found to be 9.3x10^3^ cfu/ml (or 93 cfu/10μl).

### Metagenomic analyses

#### Sequencing

Three *C*. *ramosus* larval samples (LCP2A, LCP2E, LCP2G) that were *ctxA* O1 and O139 positive were chosen for metagenomic analyses. DNA was extracted from the larvae, as was described above, and 100 ng of genomic DNA was used. For sample preparation, Swift 2S Library Kit with unique dual indexing was used (Swift Biosciences, Ann Arbor, MI). Libraries were pooled in equal volumes and initially sequenced on an Illumina MiniSeq instrument, employing a mid-output flow cell. The results of this first sequencing run (a so-called quality-control sequencing run) were used to rebalance the libraries before subsequently sequencing on an Illumina NovaSeq 6000 Sequencing System with an SP flow cell and employing paired-end 2x150 base sequencing. Library preparation and quality control sequencing were performed at the Genome Research Core at the University of Illinois at Chicago, and NovaSeq6000 sequencing was performed by the DNA Services Laboratory at the University of Illinois at Urbana-Champaign.

Metagenomic sequences were deposited in the NCBI Sequence Read Archive (SRA) (RunSelector https://www.ncbi.nlm.nih.gov/Traces/study/?acc=PRJNA604900), BioProject PRJNA604900.

### Bioinformatics analyses of metagenomic sequences

#### Taxonomic profiling

Raw reads were mapped to the NCBI nucleotide database using Centrifuge (a novel microbial classification engine) [33]. Taxonomic annotations for each read were obtained using the least-common ancestor algorithm and then summarized across all reads to create counts per taxon. Raw counts were normalized to percentages for relative abundance.

#### Functional profiling

Raw reads were mapped to the Swissprot protein database using DIAMOND [34,35]. Gene ortholog annotations were assigned using the consensus of aligned references and then summarized across all reads to create counts per ortholog for each sample. Higher level summaries of orthologous functions were created using KEGG BRITE hierarchical annotations [36]. Raw counts were normalized to percentages for relative abundance.

#### Alignment to *V*. *cholerae* references

Reference genomes and associated gene annotation files were obtained from the NCBI database. The following *V*. *cholerae* genomes were used as reference genomes: *V*. *cholerae* O1 strain 0395 (accession ID CP001235- CP001236); *V*. *cholerae* O1 biovar El Tor strain HC1037 (accession ID NZ_NGQS01000010-NZ_NGQS01000098); *V*. *cholerae* O1 strain 2010EL-1786 (ATCC BAA-2163); and *V*. *cholerae* O139 strain FC2273 (accession ID CP026647- CP026648). Raw reads were aligned to the reference genomes using BWA MEM [37]. Abundances of genes were quantitated using FeatureCounts [38] and then summarized for each contig and reference strain.

#### Data analysis

Only bacterial domain sequences were chosen for further analysis, and *V*. *cholerae* sequences were sorted. The presence of the pathogenic genomic genes from the four *V*. *cholerae* ortholog genomes (specified above) was analyzed by the annotated count file in Microsoft Excel 2019. Furthermore, virulence genes from *V*. *cholerae* O1 strain O395 (accession ID CP001235- CP001236), were searched in the metagenomic results and a map of chromosome I and II of *V*. *cholerae* was drawn using SnapGene viewer version 4.2.11.

### Statistical analyses

We applied goodness-of-fit chi-square tests to study the distribution of the prevalence of *V*. *cholerae* serogroups O1 and O139 across different chironomid species. We used two-way chi-square tests to analyze the distributions of chironomid species between the two river’s sampling points. These tests were also used to analyze the tendencies of the virulence genes (*ompW* and *ctxA*) prevalence, and *V*. *cholerae* toxigenic strains (O1 and O139) between environmental and laboratory-reared chironomids.

## Results

### Chironomid species identification and distribution

**Fig 1:**
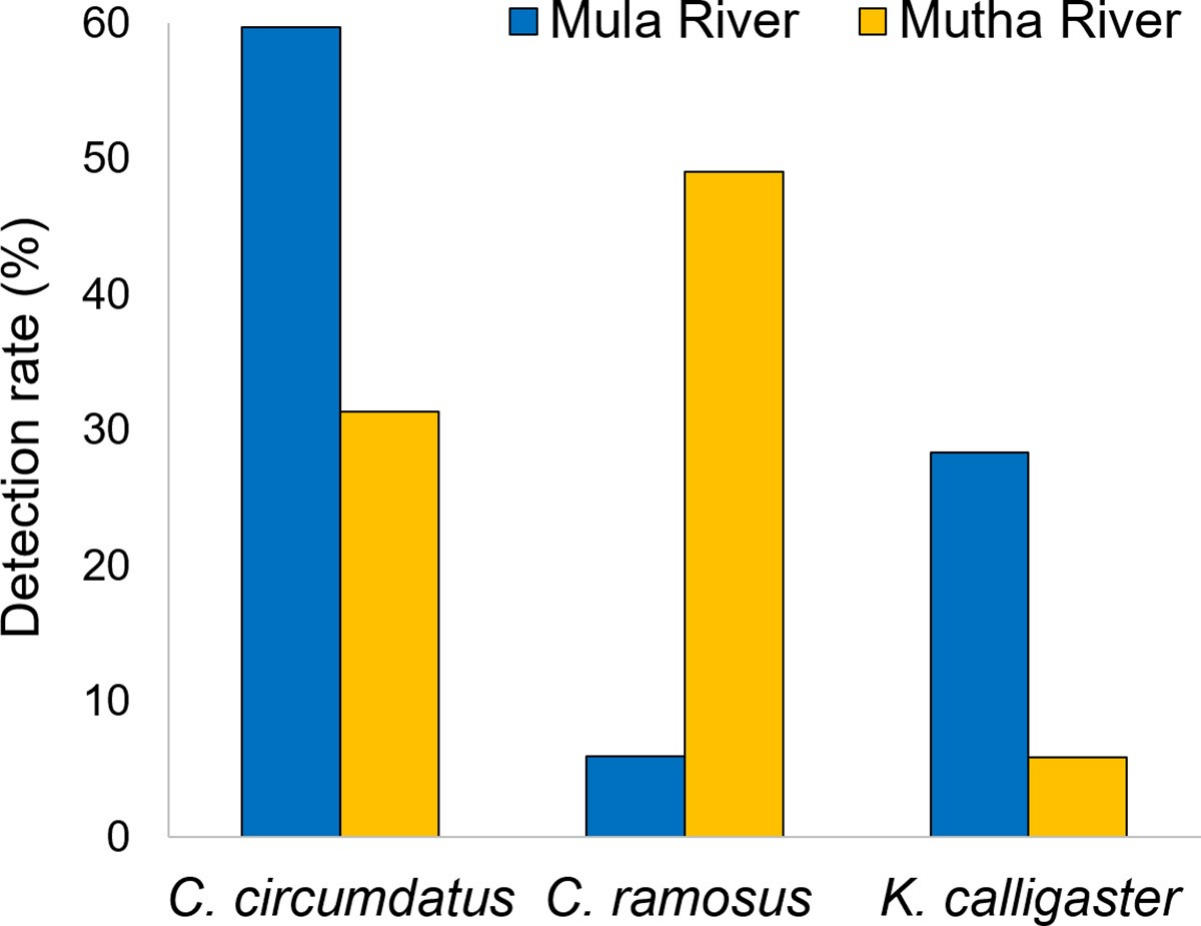
Chironomid species relative abundance (%) in Mula and Mutha Rivers, India.

Chironomids were sampled in four different sampling locations: three sampling locations were in Pune, India (i) Mula River (ii) Mutha River, and (iii) Laboratory culture. The fourth location was the Yokneam waste stabilization pond, Israel. Overall, six different chironomid species were identified from the sampling sites at the two rivers and the laboratory culture in India, while three other species were identified from the sampling site in Israel (Table 1). Two-way chi-square tests showed a significant correlation between the two river’s sampling sites and the composition of chironomid species inhabiting them. Mula River was mainly inhabited by *C*. *circumdatus* and *Kiefferulus calligaster*, with significantly higher abundances (59.7% and 28.3%, respectively) than would be expected given a random spatial distribution of these species (X^2^_2_ = 34.854, p<0.001). *C*. *ramosus*, on the other hand, was more frequently present in Mutha River (49.0%), while only four samples (5.9%) in Mula River contained this species (Fig 1 and Table 1). More species with minor abundances have been listed in Table 1.

**Table 1:** The distribution of chironomid species identified from all the investigated sampling sites. Chironomids were randomly sampled at different life stages. Adults emerged from pupae when brought to the laboratory after sampling. *C*., *Chironomus; K*., *Kiefferulus*.

Sample origin	Country		Species	Life stage	n	Sum of all life stages per species
Environmental samples	India	Mula River	*C*. *circumdatus*	Egg mass	12	40
				Larva	19	
				Pupa	9	
			*C*. *ramosus*	Egg mass	1	4
				Larva	3	
			*K*. *calligaster*	Larva	16	19
				Pupa	3	
			*K*. *tainanus*	Larva	3	4
				Pupa	1	
		Mutha River	*C*. *circumdatus*	Egg mass	7	16
				Larva	4	
				Pupa	5	
			*C*. *ramosus*	Larva	24	25
				Pupa	1	
			*C*. *kiiensis*	Egg mass	3	5
				Larva	2	
			*C*. *striatipennis*	Larva	2	2
			*K*. *calligaster*	Pupa	3	3
	Israel	Yokneam	*C*. *transvaalensis*	Egg mass	10	32
				Larva	8	
				Pupa	8	
				Adult	6	
			*K*. *brevibucca*	Adult	4	4
			*K*. sp. Israel* *	Larva	7	8
				Pupa	1	
Environmental samples that were incubated in the laboratory for eight months	India		*C*. *circumdatus*	Egg mass	7	47
				Larva	34	
				Pupa	6	
			*C*. *ramosus*	Egg mass	3	12
				Larva	9	
			*K*. *calligaster*	Pupa	2	2
**Total**						**223**

Only three chironomid species were identified in the laboratory culture that was grown from the samples taken at the Mula River eight months earlier. *C*. *circumdatus* was the dominant species, *C*. *ramosus* was the second dominant and two specimens were identified as *K*. *calligaster*. Hence, the species composition in the laboratory culture got changed compared to that of the natural source, and included mainly two species: *C*. *circumdatus* and *C*. *ramosus* (Table 1). Three chironomid species were identified in Yokneam, Israel. *C*. *transvaalensis* was the dominant species, whereas *K*. sp. Israel and *K*. *brevibucca* were less abundant (4 of 10 adults) (Table 1).

### Presence of virulence genes and toxigenic serogroups in chironomids

**Fig 2:**
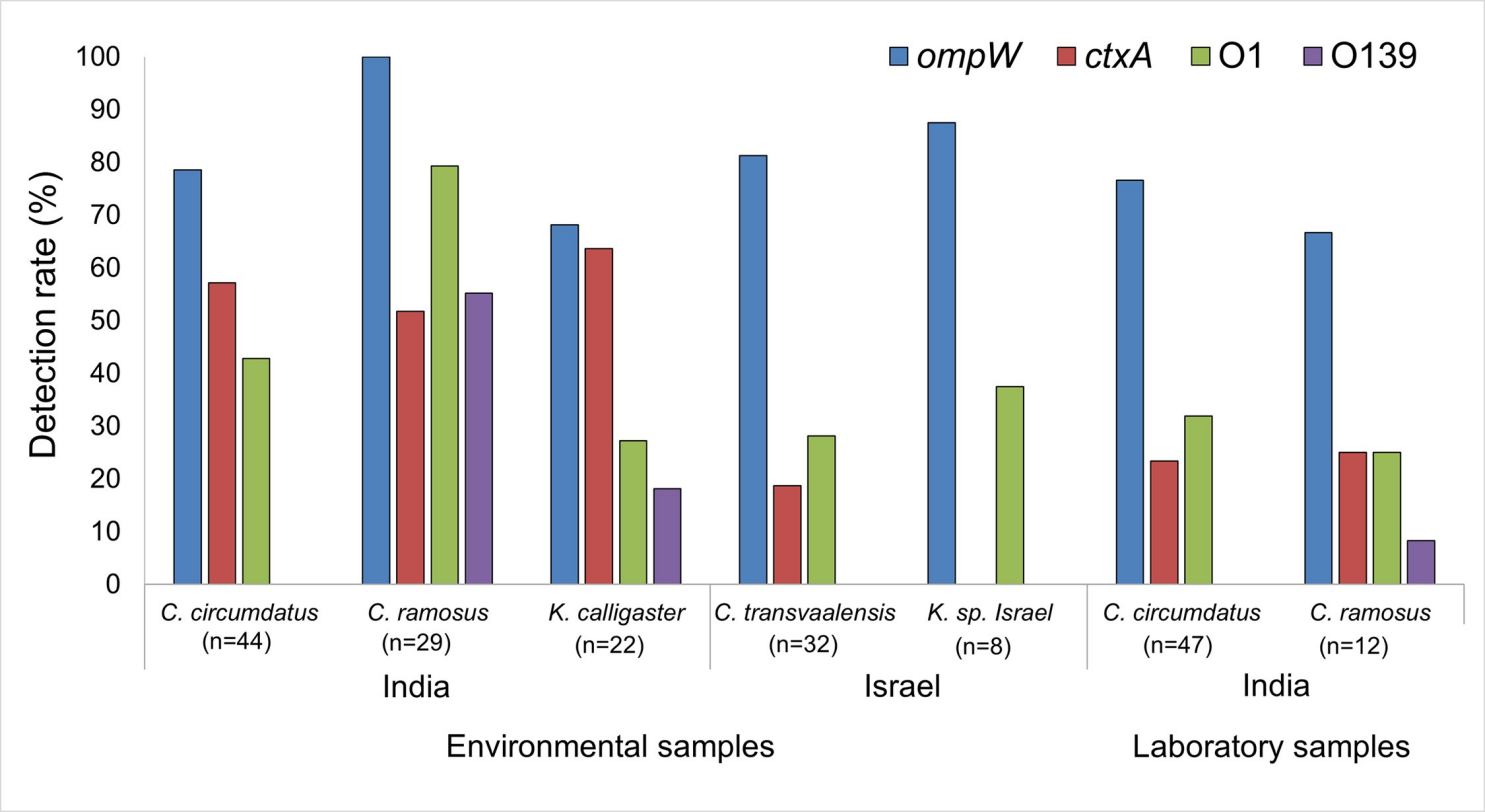
Detection rates of virulence genes and toxigenic *V*. *cholerae* serogroups at all sampling sites. To increase clarity, specimens of species with two or fewer individuals were not included in the figures and calculations. n, the total number of individuals.

Most of the environmental samples that were identified as *Chironomus* species were found to be positive for the presence of *V*. *cholerae* (that is, 86.1% of the samples were *ompW* positive). The prevalence of the *ctxA* gene in environmental samples of *C*. *circumdatus* and *C*. *ramosus* in India was 57.1% and 51.7%, respectively (Fig 2). Most environmental samples in India that were identified as *K*. *calligaster* were also positive for *ompW* and *ctxA* (68.2% and 63.6%, respectively). No significant differences were detected regarding the prevalence of the *ompW* and *ctxA* between the two river’s sampling sites (X^2^_1_ = 1.704, p = 0.192). In the environmental samples from India, 79.3% of *C*. *ramosus* samples were found to harbor the O1 serogroup, while only 42.9% of *C*. *circumdatus* and 27.3% of *K*. *calligaster* samples harbored the O1 serogroup (Fig 2). Interestingly, *V*. *cholerae* O139 serogroup was detected in India only in *C*. *ramosus* and *K*. *calligaster* species.

Significant differences were observed between the frequencies of O1 and O139 serogroups identified from the different *Chironomus* species (all environmental sites pooled). Individuals belonging to the *C*. *ramosus* species were more frequently colonized by *V*. *cholerae* O1 and O139 serogroups compared to the other chironomid species (O1: X^2^_3_ = 8.215, p = 0.042; O139: X^2^_3_ = 52.48, p<0.001).

### Gene frequency in samples from India vs. Israel

The prevalence of O1 and O139 toxigenic serogroups was significantly higher in the rivers of Pune, India, compared to the sampling site in Israel (all *Chironomus* species pooled, O1: X^2^_1_ = 4.38, p = 0.036; O139: X^2^_1_ = 8.25, p = 0.004). O1 strains were detected in 52.1% of all sampled chironomids from the rivers of Pune, while the detection rate was only 29.5% in chironomids sampled from Israel. O139 strains were detected in 17.7% of all chironomids sampled in India, whereas this serogroup was not detected at all in Israel (Fig 2). The prevalence of *ompW* did not vary significantly between the two countries, with 83.2% and 84.1% prevalence in India and Israel, respectively. However, the occurrence of *ctxA* was significantly higher in India compared to Israel (X^2^_1_ = 15.48, p<0.001) (Fig 2).

### Gene frequency in different life stages

The presence of each gene and each pathogenic serogroup was individually examined in three developmental stages (eggs, larvae, and pupae) of *C*. *circumdatus*, and *C*. *transvaalensis*. No significant differences (one-way chi-square tests; p>0.05) were found in the presence of the different genes, thereby demonstrating that *V*. *cholerae* inhabit all chironomid life stages with a similar frequency (See also S1 Table).

### Gene frequency in environmental vs. laboratory-reared chironomids

**Fig 3:**
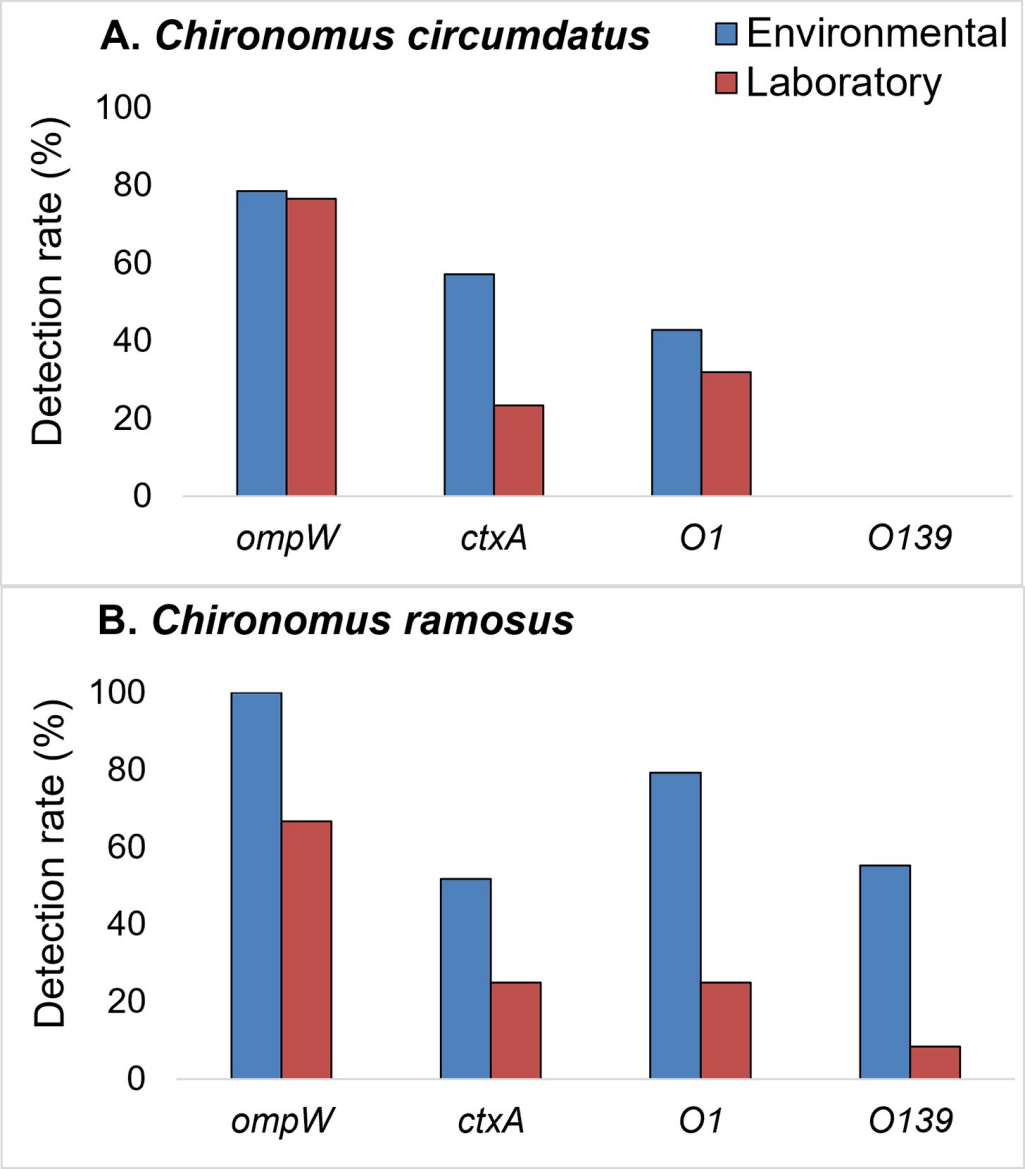
The presence of *V*. *cholerae* (*ompW*), the toxigenic strains (O1, O139), and cholera toxin subunit A (*ctxA*) in chironomid environmental samples vs. laboratory samples. The figure presents the prevalence of these genes in the two dominant *Chironomus* species (*C*. *circumdatus* and *C*. *ramosus*), identified in India.

Environmental chironomids harbored toxigenic *V*. *cholerae* strains and virulence genes at significantly higher frequencies compared to laboratory-reared chironomids. The cholera-toxin *ctxA* gene, was detected in 57.1% of the environmental *C*. *circumdatus* individuals, compared to only 23.4% of their laboratory-grown counterparts (X^2^_1_ = 11.96, p = 0.001) (Fig 3A). Similarly, *ompW* and *ctxA* were detected in 100% and 51.7%, respectively, of environmental *C*. *ramosus* samples, compared to only 66.6% and 25%, respectively, in the laboratory samples (Fig 3B). This phenomenon was also observed for the presence of O1 serogroup in *C*. *circumdatus*, wherein O1 frequency was reduced from 42.9% to 31.9% in the laboratory culture (Fig 3A). *C*. *ramosus* and *K*. *calligaster* were the only chironomid species that were inhabited by *V*. *cholerae* O139 (Fig 2). The presence of *V*. *cholerae* O1 and O139 in *C*. *ramosus* was reduced from 79.3% and 55.2%, respectively, in the environmental samples to 25% and 8.3%, respectively, under laboratory conditions (Fig 3B).

### Evidence for *V*. *cholerae* virulence genes in *C*. *ramosus* larvae

Metagenomic analysis was performed on three *C*. *ramosus* larvae that were sampled from Mutha River. Only about 6.7% of all the metagenomic sequences were related to the Bacteria domain, out of which, the overall prevalence of *Vibrio* OTUs was 0.71%. The genomes of four toxigenic *V*. *cholerae* strains (see methods) were used to detect the presence of *V*. *cholerae* virulence genes in the larvae. About 60% of the genes from the above-mentioned *V*. *cholerae* strains were detected in the larval microbiome. Table 2 and S3 Fig list those virulence genes related to the ability of *V*. *cholerae* to cause the cholera disease that we detected in metagenomic analyses. Most of these genes are in chromosome I and some are in chromosome II. The fact that these genes were present in the larval microbiome demonstrates that toxigenic *V*. *cholerae* strains indeed inhabit chironomid species.

**Table 2:** Virulence genes of *V*. *cholerae* that were detected in the metagenomic analyses of the three studied *C*. *ramosus* larvae. For each group of genes, the definition of the genes and Kyoto Encyclopedia of Genes and Genomes (KEGG) numbers (when available), are shown. Most of these genes are in chromosome I and some are in chromosome II. See also S3 Fig KEGG is a database for identifying high-level functions and utilities of biological systems [36]. *For genes that lack a KEGG number, the protein ID is given instead.

Gene	Definition	KEGG
*tagA*	N-acetylglucosaminyldiphosphoundecaprenol N-acetyl-beta-D-mannosaminyltransferase (ToxR regulated gene)	K05946
*acfA*, *B*, *C*	Accessory colonization factor	K10936; K10937; K10938
*rtxA*, *D*	RTX toxin Rtx; membrane fusion protein, RTX toxin transport system	K10953; K12532
*aldA-1*	Aldehyde dehydrogenase (ToxR regulated gene)	K00128
*ctxA*, *B*	Cholera enterotoxin subunit A, B	K10928: K10929
*mshA*, *B*, *E*	MSH pilin protein (type 4 pilus)	K10924; K10925; K12276
*rstA2*, *B2*, *R*	Cryptic phage CTXphi transcriptional repressor	K07661; T00034* (ACP09580.1; ACP09585.1)
*ompT*, *U*	Outer membrane protein	K10940; K08720
*epsC*, *D*, *E*, *F*, *H*, *I*, *K*, *L*, *M*	General secretion pathway protein (type 2 secretion system)	K02452; K02453; K02454; K02455; K02457; K02458; K02460; K02461; K02462
*Zot*	Zona occludens toxin (secretion toxin)	K10954
*pilA*, *D*	Fimbrial protein; leader peptidase (encodes type IV pilus)	K02650; K02654
*hapR*	Vibriolysin; hemagglutinin/protease regulatory protein	K08604; K10913
*hap*	hemagglutinin/protease; vibriolysin	K08604
*aceA*, *B*, *E*, *F*	Accessory cholera enterotoxin (ToxR regulated genes)	K10952
*nanH*	Sialidase, neuraminidase	K23550
*toxR*, *S*	Cholera toxin transcriptional activator; transmembrane regulatory protein	K10921; K10922
*cri-1*	Phage (ctx) replication protein	T00520* (ACP09591.1)
*pyrD*	Dihydroorotate dehydrogenase	K00254
*tcpH*, *P*	Toxin coregulated pilus biosynthesis protein	K10919; K10920
*hylB*	Hemolysin secretion protein	T00520* (ACP09591.1)

A search for the presence of genes that are related to antibiotic resistance in *V*. *cholerae* was also performed for the metagenomic sequences. The following sporadic genes were detected; *int* (integrase), *rumA* (methyltransferase, UV repair), *rumB* (methyltransferase, UV repair DNA polymerase), *dsbC* (disulfide isomerase), *traC* (type IV secretion system protein, sex pilus assembly), *ssb* (single-strand DNA binding protein), *bet* (DNA recombination protein), *radC* (DNA repair protein), *tnpA* (transposase), *floR* (chloramphenicol resistance protein), *strA* (aminoglycoside resistance protein A, streptomycin phosphotransferase), and, *strB* (streptomycin phosphotransferase). No integrating conjugative elements (ICEs) were found.

## Discussion

Chironomids were reported as the natural reservoirs of *V*. *cholerae* non-O1/O139 serogroups by Broza and Halpern in 2001 [7]. However, so far, O1 and O139 serogroups were not identified among *V*. *cholerae* isolates in this habitat [17]. In the current study, using molecular tools, we were able to identify *V*. *cholerae* O1 and the cholera toxin genes in different chironomid species that were sampled from two rivers in Pune, India, and a waste stabilization pond in northern Israel. *V*. *cholerae* O139 was also detected, but only in two chironomid species in India: *C*. *ramosus* and *K*. *calligaster*. O1 and O139 serogroups and the cholera enterotoxin subunit A (*ctxA*) gene were detected in all the life stages of the midge.

Using the fluorescence in situ hybridization (FISH) technique, Halpern et al. [9] found that an average of 3.9 X 10^4^ viable *V*. cholerae cells inhabit chironomid egg masses. The PCR limit for detecting the toxigenic genes in the current study was about 9.3 X 10^3^ *V*. *cholerae* cells per sample, thus, indicating that each positive sample had at least ca. 10^4^ *V*. *cholerae* cells per specimen. Nevertheless, the PCR method has a limitation because it detects both viable and non-viable cells.

The results of our metagenomic analysis of three *C*. *ramosus* larvae strengthened the evidence that chironomid species harbor toxigenic *V*. *cholerae* strains. A metagenomic study of microbial pathogens in water samples from Little Bighorn River in Montana, detected *V*. *cholerae* serogroup O1 biotype El Tor [39]; however, they were not able to identify the genes for cholera toxin. In contrast, in the current metagenomic analysis, we have identified both *ctxA* and *ctxB* genes in the microbiome of *C*. *ramosus* larvae. This is the first report of the presence of toxigenic strains of *V*. *cholerae* in chironomid midges which constitute an important ecological component of the freshwater aquatic food chain.

Antimicrobial resistances pose a considerable public health problem [40]. The presence of a few genes that are related to the antimicrobial resistance of *V*. *cholerae* strains was detected in the metagenomic analysis. No indication was found regarding the presence of ICEs. ICEs are transferred between two bacterial cells via conjugation and are integrated into the chromosome [41]. The first ICE that was described for *V*. *cholerae*, was the SXT element that harbors genes with resistance to sulfamethoxazole, trimethoprim, and streptomycin [42]. Currently, different O1 and O139 isolates that acquire the SXT elements are detected [43]. Kumar et al. [44] found 100% identity in a partial sequence of SXT integrase between a strain from India and a strain from Haiti, demonstrating that *V*. *cholerae* strains disseminate between continents.

In the environmental samples of *C*. *circumdatus* and *K*. *calligaster*, the prevalence of *ctxA* gene was higher compared to the prevalence of the toxigenic serogroups. Non-O1/O139 *V*. *cholerae* strains were found to possess the cholera toxin genes (e.g. *ctxA*) and have also been associated with occasional outbreaks of cholera [45,46], which can explain the relatively higher prevalence of the *ctxA* gene.

Maharashtra is classified as an endemic region for cholera. A cholera outbreak in Aurangabad, Maharashtra, was reported in November 2017 [47]. Chironomid samples in the current study were collected from Mutha and Mula Rivers in Pune, India. As far as we know, at the period of sampling (November 2018), no cholera cases were reported in Pune. This is not surprising since the citizens of Pune get uncontaminated water from the Khadakwasla dam on River Mutha. Here we present evidence that chironomids serve as natural reservoirs for the toxigenic *V*. *cholerae* O1/O139 serogroups. To confirm the connection between the presence of toxigenic *V*. *cholerae* strains in chironomids and cholera cases, an annual sampling of chironomids must be performed in the Bengal Delta region, where people drink water directly from Ganga River or from open wells, and cholera outbreaks occur at least twice a year [48].

Historically, the serogroup O139 was discovered in 1992 when a new pathogenic biotype, related to O1 El Tor, appeared in India and Bangladesh. Subsequently, this strain was named O139 and was found to have a limited distribution in the Indian subcontinent and Asia [49]. In the current study, *V*. *cholerae* O139 was identified from two species in India: *C*. *ramosus* and *K*. *calligaster*. According to the Barcode of Life Data System (BOLD) (http://v3.boldsystems.org/index.php/Taxbrowser_Taxonpage?taxid=1882), the distribution of these two chironomid species is limited to the Indian subcontinent and South Asia, as was found for the O139 serogroup. We hypothesize that one of the reasons for this limited distribution of O139 serogroup strains, may be due to the limited distribution of their chironomid species reservoirs; *C*. *ramosus* and *K*. *calligaster*. As far as we know, there is no data in the scientific literature that presents an explanation for the restricted environmental dissemination of serogroup O139.

After eight months of laboratory culture rearing, it seems that chironomid species composition changed compared to their natural habitat (Mula River). *C*. *circumdatus* and *C*. *ramosus* were identified in the laboratory culture. *K*. *calligaster* was also identified but only in two out of 47 specimens. Chironomids from the natural habitats harbored *V*. *cholerae* toxigenic strains and possessed virulence genes at significantly higher frequencies compared to laboratory-grown chironomids. Specific environmental conditions may have selected and favored chironomids harboring toxigenic *V*. *cholerae* serogroups. Thus, it appears that under laboratory conditions, chironomids lose their toxigenic *V*. *cholerae* strains. However, as the laboratory culture was collected at a different sampling period (April 2018), this may have affected the differences in *V*. *cholerae* abundances in the chironomids' laboratory culture. A laboratory experimental setup that will monitor the toxigenic genes along with at least 10 chironomid generations, may provide an answer to the hypothesis that laboratory conditions may cause chironomids to lose their toxigenic *V*. *cholerae* strains. The cholera toxin has a role in causing the disease symptoms in humans. However, its role in the environment is not clear [50]. It must confer an added advantage for the environmental survival of *V*. *cholerae* or of its host, otherwise, the environmental strains would not have retained this gene. Sakib et al. [50] hypothesized that the cholera toxin may act as an osmoregulator and thus provide an advantage in increased salinity for its host.

In the Bengal Delta region, cholera epidemics show a pattern of biannual peaks (March-May and September-November) [48]. However, the mechanisms behind these seasonal peaks are not fully understood. In a yearly survey in Israel, chironomid populations and the *V*. *cholerae* in their egg masses, similarly demonstrated biannual peaks [10,51]. If a similar chronology and a connection can be validated for chironomid populations along with the toxigenic *V*. *cholerae* serogroups in the Bengal Delta region, it can provide a useful tool and strategy for monitoring chironomid populations in endemic areas as well as for predicting cholera outbreaks. Moreover, controlling chironomid populations in the epidemic hot spots may decrease cholera cases. Thus, our study may lead to a better understanding of cholera dynamics and improve disease control.

In summary, the present study has provided evidence for the first time that nine different chironomid species from India and Israel harbor *V*. *cholerae* O1 and possess the cholera-toxin gene. Metagenomic analysis of the *C*. *ramosus* larval microbiome substantiated this evidence for the presence of *V*. *cholerae* pathogenic strains in chironomids. Only two species from India, *C*. *ramosus* and *K*. *calligaster*, harbor the toxigenic serogroup O139. The limited distribution of these two chironomid species in the Indian subcontinent and South Asia may be one of the explanations for the limited distribution of O139 serogroup. The seasonal abundance of chironomid midges together with finding evidence for the presence of toxigenic *V*. *cholerae* strains in chironomid midges and the overlapping with the peaks of cholera outbreaks in regions of epidemics, should be studied. If a positive correlation will be found between all these parameters, it may provide a tool for predicting and controlling the devastating cholera outbreaks in the future.

## Supporting information

S1 Table The prevalence of each gene and each toxigenic serogroup in the three developmental life stages (eggs, larvae, and pupae) for some of the chironomid species. No significant differences (one-way chi-square tests; p>0.05) were found between the prevalence of the different genes in the different life stages.
(PDF)

S1 Fig Environmental sampling of chironomids. **A.** Egg masses collection and separation from *Eichhornia* plants **B.** Egg masses attached to *Eichhornia crassipes* **C.** Direct sampling of chironomid larvae dislodging from their tubes **D.** Scooping larval samples by hand-held.
(TIF)

S2 Fig Chironomid rearing culture. **A.** Plastic tubs (35 cm diameter) used for rearing chironomids in the laboratory. **B.** A laboratory culture tray in a cage.
(TIF)

S3 Fig **A map of genes related to the cholera pathogenicity of *V*. *cholerae* O1** strain O395 [chromosome I (left), and chromosome II (right)]. Genes that were identified in the metagenomic analyses of three *C*. *ramosus* larval samples are listed in the Figure. The definition of each gene is specified in Table 2. The Figure was generated by SnapGene viewer version 4.2.11.
(TIF)
