# Supplementary material for: Identification of chironomid species as natural reservoirs of toxigenic Vibrio cholerae strains with pandemic potential
Source: PLoS Negl Trop Dis. 2020 Dec 23;14(12):e0008959. doi: 10.1371/journal.pntd.0008959 (PMC7757795; doi:10.1371/journal.pntd.0008959)
Supplement: S1 Table — No significant differences (one-way chi-square tests; p>0.05) were found between the prevalence of the different genes in the different life stages. (PDF) [file pntd.0008959.s001.pdf]

Supplementary Table S1. The prevalence of each gene and each toxigenic serogroup in the three developmental life stages (eggs, larvae, and pupae) for some of the chironomid species. No significant differences (one-way chi-square tests; p>0.05) were found between the prevalence of the different genes in the different life stages.

Country	Species	Life stage	*ompW*	*ctxA*	Serogroup O1	Serogroup O139	n	number of samples per species
India	*C. circumdatus*	Egg mass	0.84	0.79	0.53	0.00	**19**	**56**
		Larva	0.69	0.39	0.39	0.00	**23**	
		Pupa	0.86	0.57	0.36	0.00	**14**	
		**Average**	0.80 ± 0.05	0.58 ± 0.12	0.43 ± 0.05	0.00		
	K. calligaster	Larva	0.62	0.62	0.25	0.12	**16**	**22**
		Pupa	0.83	0.66	0.33	0.33	**6**	
		**Average**	0.73 ± 0.11	0.64 ± 0.02	0.29 ± 0.04	0.23 ± 0.11		
Israel	*C. transvaalensis*	Egg mass	0.70	0.10	0.10	0.00	**10**	**26**
		Larva	0.87	0.25	0.25	0.00	**8**	
		Pupa	0.87	0.37	0.62	0.00	**8**	
		**Average**	0.81 ± 0.06	0.24 ± 0.08	0.32 ± 0.15	0.00		
