# Supplementary figures and images for: Identification of chironomid species as natural reservoirs of toxigenic Vibrio cholerae strains with pandemic potential
Source: PLoS Negl Trop Dis. 2020 Dec 23;14(12):e0008959. doi: 10.1371/journal.pntd.0008959 (PMC7757795; doi:10.1371/journal.pntd.0008959)

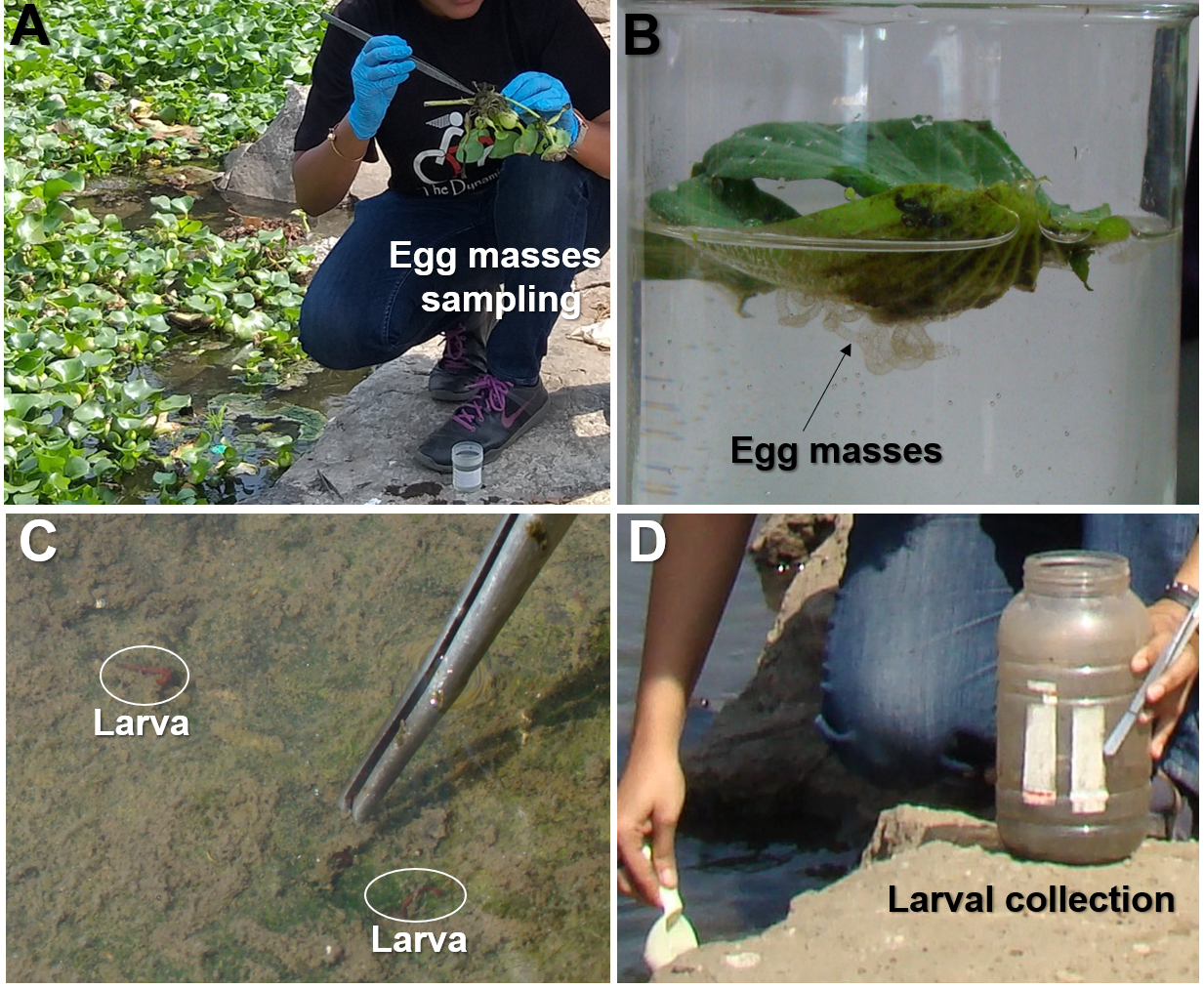

Supplement: S1 Fig — A. Egg masses collection and separation from Eichhornia plants B. Egg masses attached to Eichhornia crassipes C. Direct sampling of chironomid larvae dislodging from their tubes D. Scooping larval samples by hand-held. (TIF) [file pntd.0008959.s002.tif]

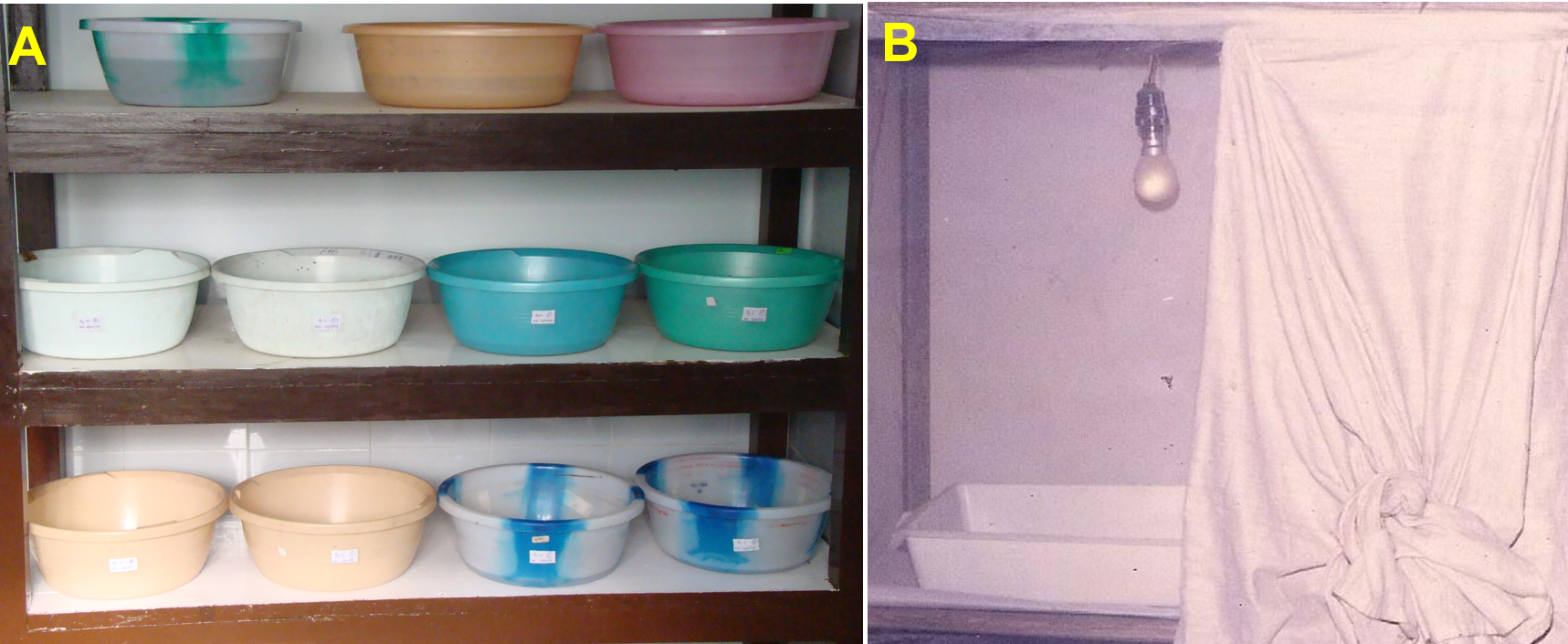

Supplement: S2 Fig — A. Plastic tubs (35 cm diameter) used for rearing chironomids in the laboratory. B. A laboratory culture tray in a cage. (TIF) [file pntd.0008959.s003.tif]

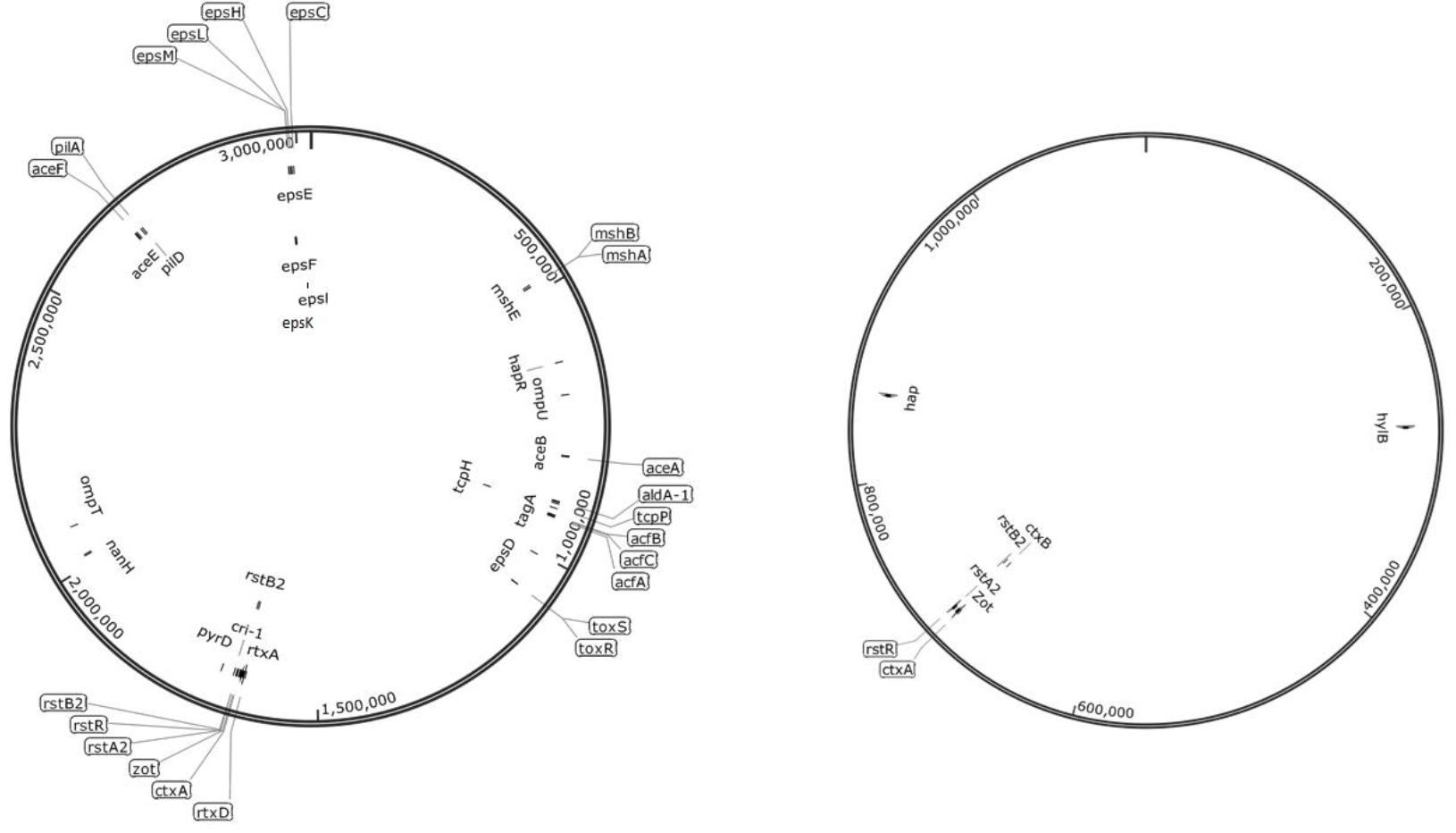

Supplement: S3 Fig — A map of genes related to the cholera pathogenicity of V. cholerae O1 strain O395 [chromosome I (left), and chromosome II (right)]. Genes that were identified in the metagenomic analyses of three C. ramosus larval samples are listed in the Figure. The definition of each gene is specified in Table 2. The Figure was generated by SnapGene viewer version 4.2.11. (TIF) [file pntd.0008959.s004.tif]
